# α-Actinin 4 Links Vasopressin Short-Term and Long-Term Regulation of Aquaporin-2 in Kidney Collecting Duct Cells

**DOI:** 10.3389/fphys.2021.725172

**Published:** 2021-12-02

**Authors:** Cheng-Hsuan Ho, Hsiu-Hui Yang, Shih-Han Su, Ai-Hsin Yeh, Ming-Jiun Yu

**Affiliations:** College of Medicine, Institute of Biochemistry and Molecular Biology, National Taiwan University, Taipei, Taiwan

**Keywords:** vasopressin, aquaporin-2, glucocorticoid receptor, collecting duct, α-actinin 4

## Abstract

Water permeability of the kidney collecting ducts is regulated by the peptide hormone vasopressin. Between minutes and hours (short-term), vasopressin induces trafficking of the water channel protein aquaporin-2 to the apical plasma membrane of the collecting duct principal cells to increase water permeability. Between hours and days (long-term), vasopressin induces aquaporin-2 gene expression. Here, we investigated the mechanisms that bridge the short-term and long-term vasopressin-mediated aquaporin-2 regulation by α-actinin 4, an F-actin crosslinking protein and a transcription co-activator of the glucocorticoid receptor. Vasopressin induced F-actin depolymerization and α-actinin 4 nuclear translocation in the mpkCCD collecting duct cell model. Co-immunoprecipitation followed by immunoblotting showed increased interaction between α-actinin 4 and glucocorticoid receptor in response to vasopressin. ChIP-PCR showed results consistent with α-actinin 4 and glucocorticoid receptor binding to the aquaporin-2 promoter. α-actinin 4 knockdown reduced vasopressin-induced increases in aquaporin-2 mRNA and protein expression. α-actinin 4 knockdown did not affect vasopressin-induced glucocorticoid receptor nuclear translocation, suggesting independent mechanisms of vasopressin-induced nuclear translocation of α-actinin 4 and glucocorticoid receptor. Glucocorticoid receptor knockdown profoundly reduced vasopressin-induced increases in aquaporin-2 mRNA and protein expression. In the absence of glucocorticoid analog dexamethasone, vasopressin-induced increases in glucocorticoid receptor nuclear translocation and aquaporin-2 mRNA were greatly reduced. α-actinin 4 knockdown further reduced vasopressin-induced increase in aquaporin-2 mRNA in the absence of dexamethasone. We conclude that glucocorticoid receptor plays a major role in vasopressin-induced aquaporin-2 gene expression that can be enhanced by α-actinin 4. In the absence of vasopressin, α-actinin 4 crosslinks F-actin underneath the apical plasma membrane, impeding aquaporin-2 membrane insertion. Vasopressin-induced F-actin depolymerization in one hand facilitates aquaporin-2 apical membrane insertion and in the other hand frees α-actinin 4 to enter the nucleus where it binds glucocorticoid receptor to enhance aquaporin-2 gene expression.

## Introduction

Vasopressin is a peptide hormone that regulates osmotic water reabsorption by the kidney collecting ducts (Knepper et al., 2015). It does so by elevating intracellular cAMP and Ca^2+^ concentrations that control the molecular water channel protein aquaporin-2 (AQP2) chiefly in two modes (Judith Radin et al., 2012; Pearce et al., 2015). Within minutes and hours (short-term), vasopressin induces AQP2 redistribution from the intracellular vesicles to the apical plasma membrane of the collecting duct principal cells to increase water permeability (Nielsen et al., 1995; Yamamoto et al., 1995). Within hours and days (long-term), vasopressin increases *AQP2* gene expression (DiGiovanni et al., 1994). Dysregulations in either regulatory mode cause a number of water balance disorders (Noda et al., 2010; Judith Radin et al., 2012; Moeller et al., 2013).

Long before the cloning of the *AQP2* gene (Fushimi et al., 1993), it has been known that the actions of vasopressin involve cytoskeleton and shuttling of intracellular membrane particles that contain transmembrane water channels (Taylor et al., 1973; Wade et al., 1981). It is now widely accepted that vasopressin depolymerizes F-actin to facilitate insertion of AQP2-containing intracellular vesicles to the apical plasma membrane of the kidney collecting duct principal cells (Simon et al., 1993; Nielsen et al., 1995; Noda et al., 2010; Fenton et al., 2020). Vasopressin-induced F-actin depolymerization was recorded in live mpkCCD cells (Loo et al., 2013), a collecting duct principal cell model that expresses all necessary molecular components required for the vasopressin actions (Yu et al., 2009). To add another layer of complexity, AQP2 constantly exocytoses to and endocytoses from the plasma membrane (Brown, 2003; Tajika et al., 2005; Wang et al., 2020). As actin is recruited to the sites of endocytosis to facilitate membrane invagination (Smythe and Ayscough, 2006), reagents that promote actin depolymerization increase AQP2 in the plasma membrane (Procino et al., 2010; Li et al., 2011). Thus, trafficking of AQP2 to and from the plasma membrane is tightly coupled with F-actin dynamics.

Efforts have been made to identify components that regulate F-actin dynamics with respect to vasopressin-induced AQP2 trafficking. An earlier proteomics study using AQP2 as a bait identified 13 AQP2-interacting proteins involved in F-actin dynamics: actin, α-actinin 4, α-II spectrin, α-tropomyosin 5b, annexin A2 and A6, gelsolin, ionized calcium binding adapter molecule 2, myosin heavy chain non-muscle type A, myosin regulatory light chain smooth muscle isoforms 2-A and 2-B, scinderin, and signal-induced proliferation-associated gene-1 (SPA-1) (Noda et al., 2005). SPA-1 is a GTPase-activating protein for Rap1, a GTPase that promotes F-actin depolymerization when bound with GTP (Richter et al., 2007). Loss-of-function mutation or deficiency in SPA-1 impairs apical AQP2 trafficking (Noda et al., 2004). The above observations led to the idea that AQP2 might, through interactions with F-actin regulatory proteins, catalyze F-actin depolymerization thereby facilitating AQP2 apical trafficking (Yui et al., 2012).

In addition to the above proteomics study, α-actinin 4 was repetitively identified in the transcriptomes and proteomes of rat kidney collecting ducts and mpkCCD cells (Uawithya et al., 2008; Yu et al., 2008, 2009; Yang et al., 2015b). Together with a network of actin regulatory proteins, α-actinin 4 protein was found in the apical plasma membrane as well as in the nuclear fraction of the mpkCCD cells (Schenk et al., 2012; Loo et al., 2013). In response to short-term vasopressin stimulation, α-actinin 4 undergoes a reciprocal abundance change in the 200,000 xg cytosolic fraction and the 17,000 xg membrane fraction (Yang et al., 2015a), suggesting dynamic shuttling of α-actinin 4 in these subcellular fractions. In response to long-term vasopressin stimulation, α-actinin 4 protein abundance increases by 30% (Khositseth et al., 2011). Apparently, α-actinin 4 is employed by vasopressin for both short- and long-term responses.

α-actinin 4 is among the four α-actinin isoforms with ∼86% amino acid homology to α-actinin 1 (Blanchard et al., 1989; Honda, 2015). α-actinin 2 and 3 belong to the muscle group whereas α-actinin 1 and 4 belong to the non-muscle group (Honda, 2015). In general, an α-actinin protein has two calponin homology domains that form an actin binding domain at the NH_2_-terminus, follow by four spectrin repeats in the middle, and two EF-hand repeats that form a calmodulin-like domain at the COOH-terminus (Honda, 2015). α-actinin forms a dimer that bundles F-actin filaments (Honda, 2015). The muscle-type actinins mediate F-actin filament bundling and interactions with the Z-disk. The non-muscle type actinins also mediate F-actin filament bundling and interactions with the cell membranes associated with cell adhesion and migration (Honda, 2015). While the binding of the muscle α-actinins to F-actin is insensitive to Ca^2+^, the binding of the non-muscle α-actinins such as α-actinin 4 to F-actin is abolished by Ca^2+^ (Blanchard et al., 1989). In addition to F-actin binding, α-actinin 4 has been shown to serve as a transcription co-activator for a number of nuclear receptors including glucocorticoid receptor (Khurana et al., 2012; Feng et al., 2015; Honda, 2015). We recently showed that the glucocorticoid receptor agonist dexamethasone potentiates vasopressin-induced *AQP2* gene expression in the mpkCCD cells (Kuo et al., 2018). Here, we provided evidence showing that vasopressin induces α-actinin 4 dissociation from F-actin and translocation into the nucleus where it interacts with glucocorticoid receptor to enhance *AQP2* gene expression.

## Materials and Methods

### Cell Culture

The mpkCCD cells re-cloned from their original line (Duong Van Huyen et al., 1998) for the highest AQP2 expression level were maintained in DMEM/Ham’s F-12 medium (DMEM/F-12, Cat. 11320033, Thermo-Fisher, United States) containing 2% fetal bovine serum (FBS) and supplements as described previously (Yu et al., 2009). Cells between 18 and 32 passages were grown on membrane supports (Transwell^®^, 0.4 μm pore size, Corning Costar, United States) prior to the experiments. FBS and supplements except dexamethasone (Kuo et al., 2018) were removed from the medium to facilitate cell polarization (transepithelial electrical resistance > 5,000 Ωxcm^2^ measured with an EVOM2 Epithelial Volt/Ohm Meter, World Precision Instruments, United States) before the cells were exposed to the vasopressin V2 receptor-specific agonist dDAVP (1-deamino-8-D-arginine vasopressin) in the basal medium to induce endogenous AQP2 expression. In some cases, dexamethasone was omitted from the experiments to examine the effects in the absence of the glucocorticoid receptor agonist. The HEK293T cells used for packaging small hairpin RNA (shRNA)-carrying lentivirus were maintained in the DMEM medium (Cat. 12491015, Thermo-Fisher, United States) containing 10% FBS.

### Immunofluorescence Confocal Microscopy

The mpkCCD cells grown on Transwell^®^ were washed with ice-cold PBS-CM [1 mM MgCl_2_ and 0.1 mM CaCl_2_ in 1X PBS (phosphate-buffered saline), pH6.4] three times prior to fixation with 4% paraformaldehyde (in PBS-CM) for 20 min at room temperature. The cells were then washed with PBS-CM three times before treated with membrane permeabilization buffer [0.3% Triton X-100, 0.1% BSA (bovine serum albumin), and 1 mM NaN_3_ in 1X PBS] for 30 min at room temperature. To block non-specific binding, the cells were incubated with IF blocking buffer (1% BSA, 0.05% saponin, 0.2% gelatin, and 1 mM NaN_3_ in 1X PBS) for 30 min at room temperature before incubated with primary antibody [α-actinin 4 (Actn4), Cat. GTX101669 or glucocorticoid receptor (GR), Cat. GTX101120, GeneTex, Taiwan] at 4°C overnight. After washes with IF washing buffer (0.1% BSA, 0.05% saponin, 0.2% gelatin, and 1 mM NaN_3_ in 1X PBS) three times, the cells were incubated with Alexa488-conjugated secondary antibody (Cat. A21206, Invitrogen, United States) for 1 h at room temperature. Cell nuclei were stained with DAPI (4′,6-diamidino-2-phenylindole, 1 μg/mL in 1X PBS) at room temperature for 10 min. In some cases, F-actin was stained with rhodamine-conjugated phalloidin (Cat. R415, Invitrogen^TM^, United States) at room temperature for 1 h. After two washes with PBS, the cells were mounted in fluorescent mounting medium (Cat. S3023, Agilent Technologies, United States) and covered with a cover glass. Confocal images were acquired with a Zeiss LSM880 microscope and processed with the ZEN blue software. Quantification of the images was done with the Imaris software (Bitplane AG, Switzerland). For colocalization measurements of two proteins, the fluorescence signals from each protein were determined with a set threshold value based on background noise, i.e., no primary antibody (or no phalloidin) staining control. Colocalization was calculated as percentage of the voxels that are doubly positive for two proteins divided by the voxels that was positive for one protein. Between 100 and 120 cells in one image were calculated, 3 images per experiment and repeated for 3 independent experiments.

### Nucleus-Cytosol Fractionation

Cells were solubilized in 0.4 ml NC buffer (10 mM HEPES, 10 mM KCl, and 0.2 mM EDTA) containing protease inhibitor (Cat. 539134, Calbiochem, United States) and phosphatase inhibitor (Cat. 524625, Calbiochem, United States). The cell lysate was set on ice for 15 min before added with NP40 to a final concentration 0.6%. The mixture was vortexed for 20 s and spun at 1,500 ×g for 5 min at 4°C before the supernatant, i.e., the cytosolic fraction was collected. The pellet was washed with 1 ml NC buffer and centrifuged at 1,500 ×g for 5 min at 4°C. The pellet was re-suspended in 0.1 ml NC buffer and sonicated. After centrifugation at 16,000 ×g for 10 min at 4°C, the supernatant, i.e., the nuclear fraction, was collected for analysis.

### Immunoblotting

Cell proteins were dissolved in SDS protein sample buffer [50 mM Tris, 150 mM NaCl, 5 mM EDTA, 1% NP40, 0.5% sodium deoxycholate, and 0.5% SDS (sodium dodecyl sulfate), pH7.4]. Protein concentrations were measured with bicinchoninic acid (Cat. 23225, Thermo-Fisher, United States). In general, 20 μg proteins were mixed with 5X loading buffer (7.5% SDS, 30% glycerol, 50 mM Tris, pH 6.8, 200 mM dithiothreitol, and bromophenol blue a few), and separated on a 10% SDS-PAGE gel at 15mA, 160V in 1X SDS-PAGE running buffer (25 mM Tris, 192 mM glycine, 0.1% SDS) for 100 min. Separated proteins in the gel were transferred to a nitrocellulose membrane (Cat. 10600004, GE Healthcare Life Science, United States) in 1X Fairbank buffer [25 mM Tris,192 mM glycine, and 20% (V/V) methanol] with 200 mA for 1 h. The membrane was incubated on a shaker at room temperature for 1 h with blocking buffer 0.1% BSA in 1X TBS-T (20 mM Tris, 150 mM NaCl, and 0.1% Tween-20). After removal of the blocking buffer, the membrane was incubated overnight with primary antibody diluted in the blocking buffer. The antibodies were Actn4 (Cat. GTX101669), glyceraldehyde-3-phosphate dehydrogenase (Gapdh, Cat. GTX100118), GR (Cat. GTX101120), histone 2A (H2A, Cat. GTX129418) from GeneTex, Taiwan, or AQP2 (sc-9880) from Santa Cruz, United States or β-actin (Cat. A5441) from Sigma-Aldrich, United States. The next day, the membrane was washed three times (10 min each) with TBS-T on a shaker, and incubated with secondary antibody (diluted in the blocking buffer) for 1 h on a shaker at room temperature. The secondary antibodies were: IRDye 800 goat anti-rabbit (Cat. 926-32211), IRDye 800 donkey anti-goat (Cat. 926-32214), or IRDye 680 goat anti-mouse (Cat. 926-68020) from Li-Cor, United States. Finally, the membrane was washed three times with TBS-T before visualization and quantification with a near-infrared fluorescence Odyssey scanner and software (Li-Cor, United States).

### Co-immunoprecipitation

Cells were solubilized in 0.3 ml lysis buffer, i.e., 1% Triton^®^ X-100 in 1X TBS (20 mM Tris and 150 mM NaCl), containing protease inhibitor and phosphatase inhibitor and left on ice for 30 min. The lysates were centrifuged (16,000 ×g, 10 min) before the protein concentrations were measured and adjusted to 2.5 mg/ml. Anti-GR antibody (Cat. sc-393232, Santa Cruz, United States) or IgG (Cat. 213111-01, GeneTex, Taiwan) was added to 1 mg lysates and incubated for 16 h at 4°C before protein G Mag Sepharose Xtra beads (Cat. GE28-9670-70, GE Healthcare, United States) were added for further incubation at 4°C for 5 h. The beads were washed with the lysis buffer before the bound proteins were eluted in the SDS protein sample buffer for SDS-PAGE and immunoblotting.

### Chromatin Immunoprecipitation Coupled With Polymerase Chain Reaction

Chromatin immunoprecipitation (ChIP) assay was performed using the SimpleChIP Enzymatic Chromatin IP kit (Cat. 9003, Cell Signaling Technologies, United States). Briefly, mpkCCD cells were freed from the culture surface, counted, and diluted to 5 × 10^5^ cells per ml in the cell culture medium. Formaldehyde (Cat. 252549, Sigma-Aldrich, United States) was added to a final concentration (1%) to crosslink proteins with chromatin at room temperature for 10 min. After the unreacted formaldehyde was quenched with glycine (125 mM) at room temperature for 5 min, chromatin was digested with 1,500 units micrococcal nuclease at 37°C for 20 min. The cells were then briefly sonicated to lyse nuclear membranes. After a brief spin at 9,400 ×g for 10 min, the supernatant was incubated with anti-GR antibody (Cat. GTX101120, GeneTex, Taiwan), anti-Actn4 antibody (Cat. M01975, Boster, United States), or control IgG antibody (provided in the kit) at 4°C with rotation overnight. The mixtures were then incubated with beads (Cat. 9006, Cell Signaling Technologies, United States) at 4°C with rotation for 2 h. After washes, the chromatin-protein complex was eluted in the ChIP Elution Buffer (Cat. 7009, Cell Signaling Technologies, United States) at 65°C for 30 min. The supernatant was then transferred to a new tube to reverse the crosslink with 200 mM NaCl and 2 μl proteinase K (Cat. 10012, Cell Signaling Technologies, United States) at 65°C for 2 h. The chromatin DNA was then purified with a spin column provided with the kit. The polymerase chain reaction (PCR) was done with 2X TOOL Taq MasterMix polymerase (Cat. KTT-BB, Tools, Taiwan) and a primer set specific to the AQP2 promoter region (forward GCAGCTCCATGGGGTAACTG and reverse CCACCCGAAGGCCTATCAC). The PCR steps were: (1) initial denaturation (94°C, 5 min); (2) denaturation (94°C, 30 s); (3) annealing (56°C, 30 s); (4) extension (72°C, 1 min); (5) repeat (steps 2–4 for 35 cycles); and (6) final extension (72°C, 5 min).

### Small Hairpin RNA-Mediated Gene Knockdown

Small hairpin RNA (shRNA)-mediated gene knockdown was done via lentivirus-based transduction. Clones for shRNA were purchased from the National RNAi Core Facility, Academia Sinica, Taiwan: shCtrl (TRCN0000208001), shGR1 (TRCN0000238464), shGR2 (TRCN0000238463), shActn4-1 (TRCN0000090213), or shActn4-2 (TRCN0000090214). To make shRNA-carrying lentivirus, the HEK293T cells were seeded at 70% confluence in a 60-mm dish. On the day of transfection, the medium was replaced with fresh DMEM containing 1% BSA minus FBS and incubated for 30 min before transfection with a lentivirus-packaging plasmid mixture: 4 μg shRNA plasmid, 3.6 μg pCMVΔ8.91 plasmid, and 0.4 μg VSVG plasmid mixed in 250 μl Opti-MEM (Cat. 31985070, Thermo-Fisher, United States) plus 12 μl T-Pro NTR II (Cat. JT97-N002M, T-Pro Biotechnology, Taiwan). Two days after the transfection, the medium that contained lentiviral particles was collected and centrifuged at 1,200 ×g for 5 min. The supernatants that contained shRNA-carrying lentiviral particles were aliquoted and stored at –80°C until use. To knockdown genes, 6 × 10^5^ mpkCCD cells were seeded in a 60-mm dish one day before infection with 1 ml lentivirus-containing medium plus 2 ml regular medium and 24 μl polybrene (hexadimethrine bromide, Cat. H9268, Sigma-Aldrich, United States, 1 mg/ml) for one day. The infected cells were selected for stable knockdown with puromycin (Watson Biotechnology, Taiwan, 2.5 μg/ml) for two passages.

### RNA Extraction and Reverse Transcription

To each membrane support (12-mm Transwell^®^, Corning Costar, United States), 300 μl TriZOL^®^ reagent (Cat. 15596081, Invitrogen, United States) were added to lyse the cells. Total RNA was then extracted with RNA extraction kit (Cat. E1011-A, ZYMO Research, United States). About 500 ng total RNA were reverse transcribed to cDNA with oligo(dT)_20_ primer (Cat. 18418020, Invitrogen, United States) or random hexamers (Cat. N8080127, Invitrogen, United States) using SuperScript^TM^ IV First-Strand Synthesis System (Cat. 18091050, Invitrogen, United States) following the manufacturer’s instruction.

### Quantitative Polymerase Chain Reaction

Quantitative polymerase chain reaction (qPCR) was carried out with SensiFAST SYBR^®^ Hi-ROX (Cat. BIO-92005, Bioline, United States) with gene-specific primers in 8-strip qRT-PCR tubes. The primers for Actn4 were forward CCAGGAGGATGACTGGGAC and reverse GCCAGCCTTCC GAAGATGA. The primers for AQP2 were forward CCTCCTTGGGATCTATTTCA and reverse CAAACTTG CCAGTGACAACT. The primers for GR were forward GACTCCAAAGAATCCTTAGCTCC and reverse CTCCACCC CTCAGGGTTTTAT. The primers for Rplp0 (60S acidic ribosomal protein P0) were forward AGAT CGGGTACCCAACTGTT and reverse GGCCTTGACC TTTTCAGTAA. The primers span intron(s) to avoid amplification of PCR products from genomic DNA. The qPCR program was done in a thermal cycler (StepOnePlus Real-Time PCR Systems, Thermo-Fisher, United States) with the following steps: (1) polymerase activation (95°C, 3 min); (2) denaturation (95°C, 5 s); (3) annealing/extension (60°C, 30 s); (4) repeat (step 2–3, 40 cycles).

## Results

### Vasopressin Freed α-Actinin 4 From F-Actin for Nuclear Translocation

**FIGURE 1:**
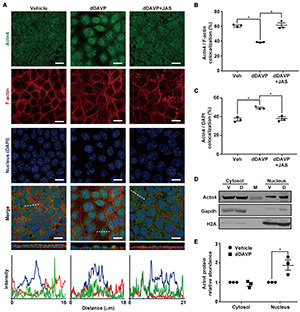
Vasopressin freed α-actinin 4 from F-actin for nuclear translocation in the mpkCCD cells. **(A)** Confocal immunofluorescence micrographs of α-actinin 4 (Actn4, green) and F-actin (red). Polarized cells grown on Transwell^®^ were exposed (24 h) to vehicle, 1 nM dDAVP, or 1 nM dDAVP plus 50 nM jasplakinolide (JAS) that binds and induces F-actin polymerization (Bubb et al., 2000). The nuclei were stained with DAPI (blue). Shown at the bottoms are signal profiles along the dotted lines. Bars indicate 10 μm. **(B,C)** Summaries of Actn4 colocalization with F-actin and DAPI, respectively. Numbers are means ± SEM, summarized from three independent experiments and about 330 cells per experiment. Asterisks indicate statistical significance, *t*-test *p* < 0.05. **(D,E)** Representative immunoblotting and summary of Actn4 in the cytosol vs. nuclei of the cells exposed to vehicle (V) vs. dDAVP (D). Numbers are means ± SEM, summarized from three independent experiments. Gapdh and H2A were markers for cytosol and nuclei, respectively. Protein intensities were corrected against those of Gapdh or H2A to avoid variations in the sample preparation. M, marker.

Under the vehicle control conditions (Figure 1A), α-actinin 4 was detected in the cytoplasm and nuclei in the polarized mpkCCD cells with confocal immunofluorescence microscopy. On average, about 60.3% α-actinin 4 was found colocalized with F-actin, which had much pronounced staining at the cell periphery (Figures 1A,B). In response to 1 nM vasopressin analog dDAVP (Figure 1A), F-actin was more diffusive with increased staining in the cytoplasm, suggestive of vasopressin-induced F-actin depolymerization. In the presence of dDAVP, less (38.5%) α-actinin 4 was found colocalized with F-actin (Figures 1A,B) and was detected primarily in the nuclei where it colocalized with DAPI that stains nucleic acids (Figure 1A). The increased nuclear α-actinin 4 staining (Figure 1C) is consistent with the idea that vasopressin frees α-actinin 4 from F-actin for nuclear translocation. In line with this, promoting F-actin polymerization with jasplakinolide significantly reduced dDAVP-induced α-actinin 4 nuclear translocation (Figures 1A–C, dDAVP + JAS). Nucleus-cytosol fractionation followed by immunoblotting showed results consistent with vasopressin-induced nuclear translocation of α-actinin 4 (Figures 1D,E).

### Vasopressin Enhanced Interaction Between α-Actinin 4 and Glucocorticoid Receptor

**FIGURE 2:**
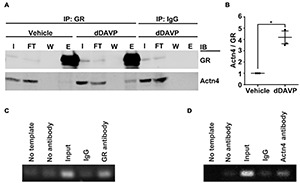
Vasopressin enhanced interactions between α-actinin 4 and glucocorticoid receptor in the mpkCCD cells. **(A)** Co-immunoprecipitation (IP) of glucocorticoid receptor (GR) followed by immunoblotting (IB) for α-actinin 4 (Actn4) in the mpkCCD cells under vehicle vs. dDAVP (1nM, 24 h) conditions. **(B)** Summary from three independent experiments. Numbers are means ± SEM, summarized from three independent experiments. Asterisk indicates statistical significance, *t*-test *p* < 0.05. E, eluate; FT, flow through; I, input; W, wash. **(C,D)** Chromatin immunoprecipitation with antibody against GR, Actn4, or IgG followed by PCR amplification of AQP2 between –128 and –255 base pairs upstream to the AQP2 transcription start site.

Co-immunoprecipitation followed by immunoblotting showed that vasopressin enhanced interaction between α-actinin 4 and glucocorticoid receptor in the mpkCCD cells. Under the vehicle conditions, there was a basal amount of α-actinin 4 in the glucocorticoid receptor immunoprecipitate (Figure 2A). In response to dDAVP, the amount of α-actinin 4 in the immunoprecipitate increased to about 4.2 folds based on three independent experiments (Figures 2A,B). Specificity of the observations was reassured with the absence of α-actinin 4 in the control IgG immunoprecipitate. In line with α-actinin 4′s function as a transcription co-activator of the glucocorticoid receptor, chromatin immunoprecipitation coupled with polymerase chain reaction showed results consistent with binding of α-actinin 4 and glucocorticoid receptor with the AQP2 promoter (Figures 2C,D). No AQP2 PCR product was observed when a control IgG was used for chromatin immunoprecipitation.

### α-Actinin 4 Knockdown Reduced Vasopressin-Induced *AQP2* Gene Expression

**FIGURE 3:**
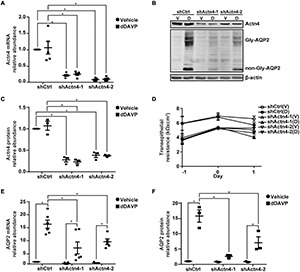
α-actinin 4 knockdown reduced vasopressin-induced *AQP2* gene expression in the mpkCCD cells. **(A,E)** Quantitative RT-PCR measurements of α-actinin 4 (Actn4) and AQP2 mRNA in control (shCtrl) and Actn4 knockdown (shActn4) cells exposed to vehicle (V) vs. 1 nM dDAVP (D). Two shActn4 sequences were used (shActn4-1 and shActn4-2). Numbers are means ± SEM, summarized from five or six independent experiments. Two-way ANOVA was used for statistical test. Asterisks indicate statistical significance between samples (*p* < 0.05). **(B,C,F)** Representative immunoblots and summaries of Actn4 and AQP2 in shCtrl and shActn4 cells in response to vehicle vs. dDAVP. Gly-AQP2 and non-Gly-AQP2 are glycosylated and non-glycosylated AQP2, respectively. Numbers are means ± SEM, summarized from three independent experiments. **(D)** Transepithelial electrical resistance of the cells grown on Transwell^®^ prior to vehicle vs. dDAVP stimulation. Day –1, cell polarization; day 0, dDAVP exposure; day 1, harvest for RNA/protein measurement.

To test α-actinin 4′s transcription co-activator function, α-actinin 4 mRNA and protein were knocked down to about 16.1% and 32.0% of the control mpkCCD cells, with two small-hairpin RNA sequences (Figures 3A–C). Vehicle or dDAVP treatment did not affect the knockdown efficiency. α-actinin 4 knockdown did not affect cell polarization as the transepithelial resistance increased in a similar way regardless experimental conditions (Figure 3D). Under these conditions, dDAVP-induced increases in the AQP2 mRNA and protein levels were greatly reduced in the α-actinin 4 knockdown vs. the control cells (Figures 3B,E,F), in line with α-actinin 4′s transcription co-activator function in vasopressin-induced *AQP2* gene expression. Since dDAVP did not affect α-actinin 4 mRNA or protein levels (Figures 3A–C), the reduced *AQP2* gene expression levels reflect the effects of the reduced α-actinin 4 levels in the knockdown cells. Note that the α-actinin 4 knockdown cells still responded to dDAVP with *AQP2* gene expression (Figures 3E,F), albeit at much reduced levels.

### Glucocorticoid Receptor Translocated Into the Nuclei in Response to Vasopressin

**FIGURE 4:**
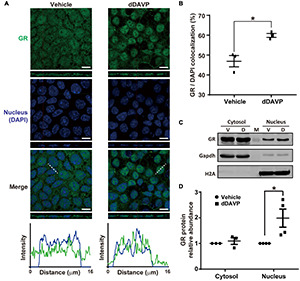
Vasopressin induced glucocorticoid receptor nuclear translocation in the mpkCCD cells in the presence of dexamethasone. **(A)** Confocal immunofluorescence micrographs of glucocorticoid receptor (GR, green) and nuclei (DAPI, blue). Polarized cells grown on Transwell^®^ were exposed (24 h) to vehicle, or 1 nM dDAVP. Shown at the bottoms are signal profiles along the dotted lines. Bars indicate 10 μm. **(B)** Summaries of GR colocalization with DAPI. Numbers are means ± SEM, summarized from three independent experiments and about 330 cells per experiment. Asterisk indicates statistical significance, *t*-test *p* < 0.05. **(C,D)** Representative immunoblots and summary of GR in the cytosol vs. nuclei of the cells exposed to vehicle (V) vs. dDAVP (D). Numbers are means ± SEM, summarized from three or four independent experiments. Gapdh and H2A were markers for cytosol and nucleus, respectively. Protein intensities were corrected against those of Gapdh or H2A to avoid variations in the sample preparation. M, marker.

Under the vehicle control conditions, glucocorticoid receptor was detected in the cytoplasm and nuclei with confocal immunofluorescence microscopy (Figure 4A). On average, about 46.9% glucocorticoid receptor was found in the nuclei where it colocalized with DAPI (Figure 4B). In response to dDAVP, more (59.9%) glucocorticoid receptor was found in the nuclei (Figures 4A,B), in line with glucocorticoid receptor nuclear translocation in response to vasopressin. Nucleus-cytosol fractionation followed by immunoblotting showed results consistent with dDAVP-induced nuclear translocation of glucocorticoid receptor (Figures 4C,D).

### Vasopressin-Induced Glucocorticoid Receptor Nuclear Translocation Was Independent of α-Actinin 4

**FIGURE 5:**
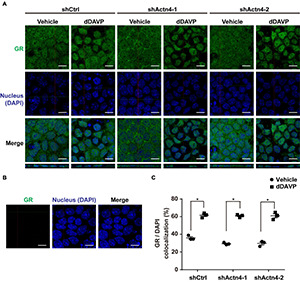
Vasopressin-induced glucocorticoid receptor nuclear translocation was independent of α-actinin 4. **(A,B)** Confocal immunofluorescence micrographs of glucocorticoid receptor in control (shCtrl) and α-actinin 4 knockdown (shActn4-1 and shActn4-2) mpkCCD cells grown on Transwell^®^ under vehicle vs. dDAVP (1 nM, 24 h) conditions. Bars indicate 10 μm. The primary antibody to glucocorticoid receptor was omitted in **(B)**. **(C)** Summary of the confocal imaging results. Numbers are means ± SEM from three independent experiments, about 330 cells in each experiment. Asterisks indicate statistical significance, *t*-test *p* < 0.05.

To test whether vasopressin-induced glucocorticoid receptor nuclear translocation depends on α-actinin 4, α-actinin 4 was knocked down before confocal immunofluorescence microscopy. Glucocorticoid receptor was detected in the cytoplasm and nuclei in the control cells under the vehicle conditions (Figure 5A, shCtrl). In response to dDAVP, more glucocorticoid receptor was observed in the nuclei than in the cytoplasm in the control cells. Similar observations were made in the α-actinin 4 knockdown cells (Figure 5A, shActn4-1 and shActn4-2). Three independent experiments show similar results (Figure 5C). Thus, vasopressin-induced glucocorticoid receptor nuclear translocation did not depend on α-actinin 4. Specificity of the observation was reassured with no primary glucocorticoid receptor antibody control, which did not produce any signal (Figure 5B).

### Glucocorticoid Receptor Knockdown Reduced Vasopressin-Induced *AQP2* Gene Expression

**FIGURE 6:**
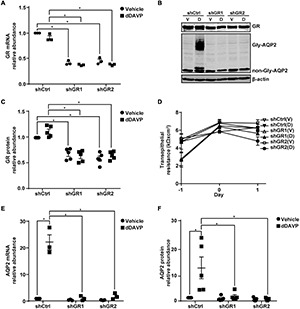
Glucocorticoid receptor knockdown reduced vasopressin-induced *AQP2* gene expression in the mpkCCD cells. **(A,E)** Quantitative RT-PCR measurements of glucocorticoid receptor (GR) and AQP2 mRNA in control (shCtrl) and GR knockdown (shGR) cells in response to vehicle vs. 1 nM dDAVP. Two shGR sequences were used (shGR1 and shGR2). Numbers are means ± SEM, summarized from three independent experiments. Two-way ANOVA was used for statistical test. Asterisks indicate statistical significance between samples (*p* < 0.05, *t*-test). **(B,C,F)** Representative immunoblots and summaries of GR and AQP2 in shCtrl and shGR cells in response to vehicle (V) vs. dDAVP (D). Gly-AQP2 and non-Gly-AQP2 are glycosylated and non-glycosylated AQP2, respectively. Numbers are means ± SEM, summarized from five independent experiments. **(D)** Transepithelial electrical resistance of the cells grown on Transwell^®^ prior to vehicle vs. dDAVP stimulation. Day –1, cell polarization; day 0, dDAVP exposure; day 1, harvest for RNA and protein measurements.

Glucocorticoid receptor mRNA and protein knockdown to about 40.9% or 63.1% (Figures 6A–C) did not affect mpkCCD cell polarization as the increases in the transepithelial resistance were similar for all experimental groups (Figure 6D). Under these conditions, the dDAVP-induced increases in the *AQP2* gene expression were profoundly reduced at both mRNA and protein levels in the glucocorticoid receptor knockdown cells (Figures 6B,E,F). dDAVP did not affect glucocorticoid receptor mRNA or protein levels in the control or knockdown cells exposed to vehicle or dDAVP (Figures 6A–C). Thus, the reduced *AQP2* gene expression were due to the effects of the reduced glucocorticoid receptor levels in the knockdown cells. Note that while the AQP2 mRNA and protein were detected in the α-actinin 4 knockdown cells (Figures 3B,E,F), they were barely detectable in the glucocorticoid receptor knockdown cells (Figures 6B,E,F), indicating a major role of glucocorticoid receptor in vasopressin-induced *AQP2* gene expression.

### Vasopressin-Induced Glucocorticoid Receptor Nuclear Translocation and AQP2 mRNA Expression Were Reduced in the Absence of Dexamethasone

**FIGURE 7:**
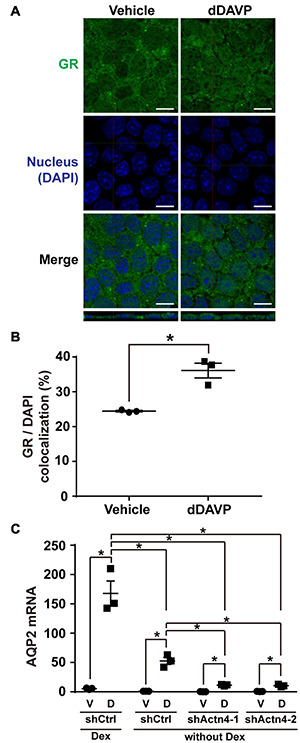
Vasopressin-induced glucocorticoid receptor nuclear translocation and AQP2 mRNA expression were reduced in the absence of dexamethasone. **(A,B)** Representative and summary of confocal immunofluorescence micrographs of glucocorticoid receptor (GR) in the mpkCCD cells in response to vehicle or dDAVP in the absence of dexamethasone. The nuclei were stained with DAPI in blue. Numbers are means ± SEM, summarized from three independent experiments and about 330 cells per experiment. Asterisks indicate statistical significance, *t*-test *p* < 0.05. Bars indicate 10 μm. **(C)** Quantitative RT-PCR measurements of AQP2 mRNA in the control (shCtrl) and α-actinin 4 knockdown (shActn4-1 and shActn4-2) mpkCCD cells in response to dDAVP (D) vs. vehicle (V) in the presence or absence of dexamethasone (Dex). Numbers are means ± SEM, summarized from three independent experiments.

The above experiments were done in the presence of glucocorticoid receptor agonist dexamethasone that induces glucocorticoid receptor nuclear translocation (Gustafsson et al., 1987). To test whether vasopressin-induced glucocorticoid receptor nuclear translocation and *AQP2* gene expression depends on glucocorticoid, dexamethasone was omitted from the experiments. Confocal immunofluorescence microscopy showed cytoplasmic and nuclear staining of glucocorticoid receptor in the mpkCCD cells under the vehicle conditions in the absence of dexamethasone (Figure 7A). Stimulating the cells with dDAVP in the absence of dexamethasone slightly increased nuclear staining of glucocorticoid receptor. On average, the nuclear glucocorticoid receptor staining increased from 24.4% under the vehicle conditions to 37.2% under the dDAVP conditions in the absence of dexamethasone (Figure 7B). Thus, glucocorticoid binding plays a crucial role in vasopressin-induced glucocorticoid receptor nuclear translocation. Similarly, glucocorticoid binding also plays a crucial role in vasopressin-induced *AQP2* gene expression. In the presence of dexamethasone, dDAVP induced AQP2 mRNA to about 31.0 folds compared to that in the vehicle control (Figure 7C). In the absence of dexamethasone, the dDAVP-induced AQP2 mRNA level was reduced to about 1/3 of that in the presence of dexamethasone. In the absence of dexamethasone, the dDAVP-induced AQP2 mRNA level in the α-actinin 4 knockdown cells was further reduced to about 1/5 of that in the control cells (Figure 7C, shActn4 vs. shCtrl). Thus, dexamethasone plays a major role in vasopressin-induced glucocorticoid receptor nuclear translocation and *AQP2* gene expression, which can be enhanced by α-actinin 4.

## Discussion

**FIGURE 8:**
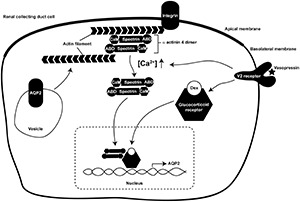
A model illustrates dual functions of α-actinin 4 in vasopressin short-term and long-term actions in the kidney collecting duct cells. ABD, actin-binding domain; CaM, calmodulin-like domain; Dex, dexamethasone.

In the present study, we showed for the first time that α-actinin 4 functions as a transcription co-activator for glucocorticoid receptor-mediated *AQP2* gene expression in the mpkCCD collecting duct cells in response to vasopressin (Kuo et al., 2018). Our findings suggest a role of α-actinin 4 that connects vasopressin-mediated short-term regulation of AQP2 trafficking to long-term regulation of *AQP2* gene expression (Figure 8). As an F-actin crosslinking protein, α-actinin 4 dimers bundle F-actin filaments and link them to the plasma membrane via direct or indirect interaction with integrin (Chen et al., 2012; Honda, 2015). This forms a cortical F-actin skeleton underneath the apical plasma membrane and impedes AQP2-containing vesicle from fusion with the membrane (Noda et al., 2010). Vasopressin-induced apical AQP2 insertion thus necessitates F-actin depolymerization at the apical plasma membrane (Noda et al., 2008; Yui et al., 2012; Loo et al., 2013). Via elevating intracellular Ca^2+^, vasopressin reduces α-actinin 4 interaction with F-actin (Blanchard et al., 1989; Knepper et al., 2015). This should free up α-actinin 4 and promote F-actin depolymerization (Star et al., 1988; Blanchard et al., 1989), which is expected to facilitate short-term vasopressin-induced AQP2 insertion into the apical membrane. The so freed α-actinin 4 then enters the nuclei where it interacts with glucocorticoid receptor to enhance long-term vasopressin-induced *AQP2* gene expression.

Functions of F-actin in AQP2 trafficking are far more complex than those described above. As AQP2 constitutively recycles between the intracellular vesicles and the plasma membrane (Brown, 2003; Tajika et al., 2005; Wang et al., 2020), the apical AQP2 abundance is a balanced result between AQP2 exocytosis to and endocytosis from the apical plasma membrane. While increased exocytosis increases the apical AQP2 abundance (Noda and Sasaki, 2005), decreased endocytosis also does so (Brown, 2003). The facts that AQP2 is endocytosed via clathrin-mediated processes (Sun et al., 2002) and that F-actin participates in near all aspects of clathrin-mediated endocytosis (Yarar et al., 2005) have alluded to functions of F-actin in AQP2 endocytosis. As F-actin is recruited to the sites of endocytosis to facilitate membrane invagination (Smythe and Ayscough, 2006), reagents that promote F-actin depolymerization reduce AQP2 endocytosis and increase AQP2 in the plasma membrane (Procino et al., 2010; Li et al., 2011). Conversely, F-actin polymerization promotes AQP2 endocytosis (Li et al., 2011, 2017). The above observations are compatible with the regulation of F-actin by α-actinin 4 in the collecting duct cells (Figure 8). In the absence of vasopressin, intracellular Ca^2+^ concentration is low, α-actinin 4 dimers bundle F-actin and connect them to the plasma membrane. This prevents AQP2 exocytosis in one hand and promotes AQP2 endocytosis in the other hand. In the presence of vasopressin, intracellular Ca^2+^ concentration elevates and reduces α-actinin 4 interaction with F-actin (Blanchard et al., 1989), promoting F-actin depolymerization. This is expected to reduce AQP2 endocytosis and to facilitate AQP2 exocytosis. Vasopressin-induced AQP2 phosphorylation further enhances apical AQP2 abundance via these two processes. S256 phosphorylation promotes F-actin depolymerization, which is expected to reduce AQP2 endocytosis and enhance exocytosis (Noda et al., 2008). S269 phosphorylation has been shown to reduce Sipa1l1-mediated AQP2 endocytosis (Wang et al., 2017).

Compared to α-actinin 4 knockdown, glucocorticoid receptor knockdown had much profound effects on vasopressin-induced *AQP2* gene expression. While AQP2 mRNA and protein were detected in the α-actinin 4 knockdown cells in the presence of vasopressin (Figure 3), they were barely detectable in the glucocorticoid receptor knockdown cells (Figure 6). This is consistent with the transcription co-activator role of α-actinin 4, which does not directly bind glucocorticoid response elements but complexes with glucocorticoid receptor to enhance transcriptional activity (Honda, 2015; Zhao et al., 2017). Note that the experiments in the present study were done in the presence of the glucocorticoid receptor agonist dexamethasone, which itself would bind glucocorticoid receptor and induce nuclear translocation. This may explain the nuclear staining of the glucocorticoid receptor in the absence of vasopressin (Figure 4). However, the nuclear dexamethasone-glucocorticoid receptor complex did not result in *AQP2* gene expression in the absence of vasopressin (Figure 6), consistent with our prior observation (Kuo et al., 2018). Thus, vasopressin must have elicited additional mechanisms to result in *AQP2* gene expression even in the presence of nuclear glucocorticoid receptor. Vasopressin-induced α-actinin 4 nuclear translocation and interaction with glucocorticoid receptor contribute a portion to the mechanism as α-actinin 4 knockdown reduces vasopressin-induced *AQP2* gene expression in the presence of dexamethasone (Figures 1–3).

How vasopressin leads to glucocorticoid receptor nuclear translocation requires further study. Vasopressin-enhanced interaction between glucocorticoid receptor and α-actinin 4 (Figure 2) does not seem to provide such a mechanism because vasopressin still induces glucocorticoid receptor nuclear translocation in the α-actinin 4 knockdown cells (Figure 5) and still induces *AQP2* gene expression (Figure 3). Moreover, α-actinin 4 itself lacks nuclear localization signal (Honda et al., 1998). How α-actinin 4 translocates to the nucleus also requires further investigation. Our data suggest two independent pathways by which glucocorticoid receptor and α-actinin 4 enter the nucleus in response to vasopressin (Figure 8). Once in the nucleus, glucocorticoid receptor and α-actinin 4 interact to enhance *AQP2* gene expression. In fact, α-actinin 4 was found to interact with glucocorticoid receptor exclusively in the nuclei in human podocytes (Zhao et al., 2017).

The dual functional α-actinin 4 provides a molecular link that connects vasopressin short-term regulation in AQP2 trafficking to long-term regulation in *AQP2* gene transcription (Figure 8); however, the network of vasopressin-mediated *AQP2* gene transcription is far from complete. Based on promoter-reporter assays, several transcription factors have been implicated in AQP2 transcription including Creb1 (Hozawa et al., 1996; Yasui et al., 1997), Elf3 (Yu et al., 2009), Elf5 (Yu et al., 2009; Grassmeyer et al., 2017), Ehf (Yu et al., 2009), Gata-3 (Uchida et al., 1997), and Nfat5 (Hasler et al., 2006). Incorporation of these transcription factors into the AQP2 transcription network is challenging because the studies were done in various cell models that often do not express endogenous vasopressin V2 receptor or AQP2. For example, Creb1 has been the primary transcription factor for *AQP2* gene transcription in many review papers (Nielsen et al., 2002; Bockenhauer and Bichet, 2015; Pearce et al., 2015). However, recent ChIP-seq analysis of the re-cloned mpkCCD cells that express all necessary molecular components for vasopressin signaling, *AQP2* gene expression, and trafficking (Rinschen et al., 2010; Xie et al., 2010; Khositseth et al., 2011; Schenk et al., 2012; Loo et al., 2013) showed no clear evidence of Creb1’s involvement in AQP2 transcription (Jung et al., 2018). Instead, a number of other transcription factor candidates were suggested for future investigation (Kikuchi et al., 2021), preferentially with CRISPR/Cas9-based gene knockout (Isobe et al., 2017, 2020; Datta et al., 2020) or small hairpin RNA-mediated gene knockdown when gene deletion is lethal (Wang et al., 2017, 2020; Kuo et al., 2018; Lin et al., 2019; Wong et al., 2020). Thus, a comprehensive AQP2 transcription network may be anticipated with the advent of the systems approaches that generate novel hypotheses addressable with modern molecular biological methods.

## Data Availability Statement

The raw data supporting the conclusions of this article will be made available by the authors, without undue reservation.

## Author Contributions

C-HH, H-HY, S-HS, and A-HY designed and performed experiments. C-HH, H-HY, S-HS, A-HY, and M-JY analyzed, presented, and interpreted the data. All authors wrote, edited, and approved the manuscript.

## Conflict of Interest

The authors declare that the research was conducted in the absence of any commercial or financial relationships that could be construed as a potential conflict of interest.

## Publisher’s Note

All claims expressed in this article are solely those of the authors and do not necessarily represent those of their affiliated organizations, or those of the publisher, the editors and the reviewers. Any product that may be evaluated in this article, or claim that may be made by its manufacturer, is not guaranteed or endorsed by the publisher.

## References

[B1] Blanchard A. Ohanian V. Critchley D. (1989). The structure and function of alpha-actinin. *J. Muscle Res. Cell Motil.* 10 280–289. 2671039 10.1007/BF01758424

[B2] Bockenhauer D. Bichet D. G. (2015). Pathophysiology, diagnosis and management of nephrogenic diabetes insipidus. *Nat. Rev. Nephrol.* 11 576–588. 10.1038/nrneph.2015.89 26077742

[B3] Brown D. (2003). The ins and outs of aquaporin-2 trafficking. *Am. J. Physiol. Renal Physiol.* 284 F893–F901. 10.1152/ajprenal.00387.2002 12676734

[B4] Bubb M. R. Spector I. Beyer B. B. Fosen K. M. (2000). Effects of jasplakinolide on the kinetics of actin polymerization. An explanation for certain *in vivo* observations. *J. Biol. Chem.* 275 5163–5170. 10.1074/jbc.275.7.5163 10671562

[B5] Chen Y. Rice W. Gu Z. Li J. Huang J. Brenner M. B. (2012). Aquaporin 2 promotes cell migration and epithelial morphogenesis. *J. Am. Soc. Nephrol.* 23 1506–1517. 10.1681/asn.2012010079 22859853 PMC3431417

[B6] Datta A. Yang C. R. Limbutara K. Chou C. L. Rinschen M. M. Raghuram V. (2020). PKA-independent vasopressin signaling in renal collecting duct. *FASEB J.* 34 6129–6146. 10.1096/fj.201902982R 32219907 PMC9200475

[B7] DiGiovanni S. R. Nielsen S. Christensen E. I. Knepper M. A. (1994). Regulation of collecting duct water channel expression by vasopressin in Brattleboro rat. *Proc. Natl. Acad. Sci. U.S.A.* 91 8984–8988. 7522327 10.1073/pnas.91.19.8984 PMC44731

[B8] Duong Van Huyen J. Bens M. Vandewalle A. (1998). Differential effects of aldosterone and vasopressin on chloride fluxes in transimmortalized mouse cortical collecting duct cells. *J. Membr. Biol.* 164 79–90. 10.1007/s002329900395 9636246

[B9] Feng D. DuMontier C. Pollak M. R. (2015). The role of alpha-actinin-4 in human kidney disease. *Cell Biosci.* 5:44. 10.1186/s13578-015-0036-8 PMC4545552 26301083

[B10] Fenton R. A. Murali S. K. Moeller H. B. (2020). Advances in Aquaporin-2 trafficking mechanisms and their implications for treatment of water balance disorders. *Am. J. Physiol. Cell Physiol.* [Epub ahead of print]. 10.1152/ajpcell.00150.2020 32432927

[B11] Fushimi K. Uchida S. Hara Y. Hirata Y. Marumo F. Sasaki S. (1993). Cloning and expression of apical membrane water channel of rat kidney collecting tubule. *Nature* 361 549–552. 8429910 10.1038/361549a0

[B12] Grassmeyer J. Mukherjee M. deRiso J. Hettinger C. Bailey M. Sinha S. (2017). Elf5 is a principal cell lineage specific transcription factor in the kidney that contributes to Aqp2 and Avpr2 gene expression. *Dev. Biol.* 424 77–89. 10.1016/j.ydbio.2017.02.007 28215940 PMC5382981

[B13] Gustafsson J. A. Carlstedt-Duke J. Poellinger L. Okret S. Wikstrom A. C. Bronnegard M. (1987). Biochemistry, molecular biology, and physiology of the glucocorticoid receptor. *Endocr. Rev.* 8 185–234. 3038508 10.1210/edrv-8-2-185

[B14] Hasler U. Jeon U. S. Kim J. A. Mordasini D. Kwon H. M. Feraille E. (2006). Tonicity-responsive enhancer binding protein is an essential regulator of aquaporin-2 expression in renal collecting duct principal cells. *J. Am. Soc. Nephrol.* 17 1521–1531. 10.1681/ASN.2005121317 16641150

[B15] Honda K. (2015). The biological role of actinin-4 (ACTN4) in malignant phenotypes of cancer. *Cell Biosci.* 5:41. 10.1186/s13578-015-0031-0 PMC4539665 26288717

[B16] Honda K. Yamada T. Endo R. Ino Y. Gotoh M. Tsuda H. (1998). Actinin-4, a novel actin-bundling protein associated with cell motility and cancer invasion. *J. Cell Biol.* 140 1383–1393. 10.1083/jcb.140.6.1383 9508771 PMC2132673

[B17] Hozawa S. Holtzman E. J. Ausiello D. A. (1996). cAMP motifs regulating transcription in the aquaporin 2 gene. *Am. J. Physiol.* 270 C1695–C1702. 8764152 10.1152/ajpcell.1996.270.6.C1695

[B18] Isobe K. Jung H. J. Yang C. R. Claxton J. Sandoval P. Burg M. B. (2017). Systems-level identification of PKA-dependent signaling in epithelial cells. *Proc. Natl. Acad. Sci. U.S.A.* 114 E8875–E8884. 28973931 10.1073/pnas.1709123114 PMC5651769

[B19] Isobe K. Raghuram V. Krishnan L. Chou C. L. Yang C. R. Knepper M. A. (2020). CRISPR-Cas9/phosphoproteomics identifies multiple noncanonical targets of myosin light chain kinase. *Am. J. Physiol. Renal Physiol.* 318 F600–F616. 10.1152/ajprenal.00431.2019 31904282 PMC7099502

[B20] Judith Radin M. Yu M. J. Stoedkilde L. Lance Miller R. Hoffert J. D. Frokiaer J. (2012). Aquaporin-2 regulation in health and disease. *Vet. Clin. Pathol.* 41 455–470. 23130944 10.1111/j.1939-165x.2012.00488.x PMC3562700

[B21] Jung H. J. Raghuram V. Lee J. W. Knepper M. A. (2018). Genome-wide mapping of DNA accessibility and binding sites for CREB and C/EBPbeta in vasopressin-sensitive collecting duct cells. *J. Am. Soc. Nephrol.* 29 1490–1500. 10.1681/ASN.2017050545 29572403 PMC5967763

[B22] Khositseth S. Pisitkun T. Slentz D. H. Wang G. Hoffert J. D. Knepper M. A. (2011). Quantitative protein and mRNA profiling shows selective post-transcriptional control of protein expression by vasopressin in kidney cells. *Mol. Cell. Proteomics* 10:M110004036. 10.1074/mcp.M110.004036 20940332 PMC3013460

[B23] Khurana S. Chakraborty S. Lam M. Liu Y. Su Y. T. Zhao X. (2012). Familial focal segmental glomerulosclerosis (FSGS)-linked alpha-actinin 4 (ACTN4) protein mutants lose ability to activate transcription by nuclear hormone receptors. *J. Biol. Chem.* 287 12027–12035. 10.1074/jbc.M112.345421 22351778 PMC3320949

[B24] Kikuchi H. Jung H. J. Raghuram V. Leo K. T. Park E. Yang C. R. (2021). Bayesian identification of candidate transcription factors for the regulation of Aqp2 gene expression. *Am. J. Physiol. Renal Physiol.* 321 F389–F401. 10.1152/ajprenal.00204.2021 34308668 PMC8530753

[B25] Knepper M. A. Kwon T. H. Nielsen S. (2015). Molecular physiology of water balance. *N. Engl. J. Med.* 372 1349–1358. 25830425 10.1056/NEJMra1404726 PMC6444926

[B26] Kuo K. T. Yang C. W. Yu M. J. (2018). Dexamethasone enhances vasopressin-induced aquaporin-2 gene expression in the mpkCCD cells. *Am. J. Physiol. Renal Physiol.* 314 F219–F229. 10.1152/ajprenal.00218.2017 29070569

[B27] Li W. Jin W. W. Tsuji K. Chen Y. Nomura N. Su L. (2017). Ezrin directly interacts with AQP2 and promotes its endocytosis. *J. Cell Sci.* 130 2914–2925. 10.1242/jcs.204842 28754689 PMC5612225

[B28] Li W. Zhang Y. Bouley R. Chen Y. Matsuzaki T. Nunes P. (2011). Simvastatin enhances aquaporin-2 surface expression and urinary concentration in vasopressin-deficient Brattleboro rats through modulation of Rho GTPase. *Am. J. Physiol. Renal Physiol.* 301 F309–F318. 10.1152/ajprenal.00001.2011 21511701 PMC3154588

[B29] Lin S. T. Ma C. C. Kuo K. T. Su Y. F. Wang W. L. Chan T. H. (2019). Transcription Factor Elf3 Modulates Vasopressin-Induced Aquaporin-2 Gene Expression in Kidney Collecting Duct Cells. *Front Physiol* 10:1308. 10.3389/fphys.2019.01308 31681015 PMC6813252

[B30] Loo C. S. Chen C. W. Wang P. J. Chen P. Y. Lin S. Y. Khoo K. H. (2013). Quantitative apical membrane proteomics reveals vasopressin-induced actin dynamics in collecting duct cells. *Proc. Natl. Acad. Sci. U.S.A.* 110 17119–17124. 10.1073/pnas.1309219110 24085853 PMC3800992

[B31] Moeller H. B. Rittig S. Fenton R. A. (2013). Nephrogenic diabetes insipidus: essential insights into the molecular background and potential therapies for treatment. *Endocr. Rev.* 34 278–301. 10.1210/er.2012-1044 23360744 PMC3610677

[B32] Nielsen S. Chou C. L. Marples D. Christensen E. I. Kishore B. K. Knepper M. A. (1995). Vasopressin increases water permeability of kidney collecting duct by inducing translocation of aquaporin-CD water channels to plasma membrane. *Proc. Natl. Acad. Sci. U.S.A.* 92 1013–1017. 10.1073/pnas.92.4.1013 7532304 PMC42627

[B33] Nielsen S. Frokiaer J. Marples D. Kwon T. H. Agre P. Knepper M. A. (2002). Aquaporins in the kidney: from molecules to medicine. *Physiol. Rev.* 82 205–244. 10.1152/physrev.00024.2001 11773613

[B34] Noda Y. Horikawa S. Furukawa T. Hirai K. Katayama Y. Asai T. (2004). Aquaporin-2 trafficking is regulated by PDZ-domain containing protein SPA-1. *FEBS Lett.* 568 139–145. 10.1016/j.febslet.2004.05.021 15196935

[B35] Noda Y. Horikawa S. Kanda E. Yamashita M. Meng H. Eto K. (2008). Reciprocal interaction with G-actin and tropomyosin is essential for aquaporin-2 trafficking. *J. Cell Biol.* 182 587–601. 10.1083/jcb.200709177 18678705 PMC2500142

[B36] Noda Y. Horikawa S. Katayama Y. Sasaki S. (2005). Identification of a multiprotein “motor” complex binding to water channel aquaporin-2. *Biochem. Biophys. Res. Commun.* 330 1041–1047. 10.1016/j.bbrc.2005.03.079 15823548

[B37] Noda Y. Sasaki S. (2005). Trafficking mechanism of water channel aquaporin-2. *Biol. Cell* 97 885–892. 10.1042/bc20040120 16293109

[B38] Noda Y. Sohara E. Ohta E. Sasaki S. (2010). Aquaporins in kidney pathophysiology. *Nat. Rev. Nephrol.* 6 168–178. 10.1038/nrneph.2009.231 20101255

[B39] Pearce D. Soundararajan R. Trimpert C. Kashlan O. B. Deen P. M. Kohan D. E. (2015). Collecting duct principal cell transport processes and their regulation. *Clin. J. Am. Soc. Nephrol.* 10 135–146. 10.2215/cjn.05760513 24875192 PMC4284417

[B40] Procino G. Barbieri C. Carmosino M. Rizzo F. Valenti G. Svelto M. (2010). Lovastatin-induced cholesterol depletion affects both apical sorting and endocytosis of aquaporin-2 in renal cells. *Am. J. Physiol. Renal Physiol.* 298 F266–F278. 10.1152/ajprenal.00359.2009 19923410

[B41] Richter M. Murai K. K. Bourgin C. Pak D. T. Pasquale E. B. (2007). The EphA4 receptor regulates neuronal morphology through SPAR-mediated inactivation of Rap GTPases. *J. Neurosci.* 27 14205–14215. 10.1523/JNEUROSCI.2746-07.2007 18094260 PMC6673515

[B42] Rinschen M. M. Yu M. J. Wang G. Boja E. S. Hoffert J. D. Pisitkun T. (2010). Quantitative phosphoproteomic analysis reveals vasopressin V2-receptor-dependent signaling pathways in renal collecting duct cells. *Proc. Natl. Acad. Sci. U.S.A.* 107 3882–3887. 10.1073/pnas.0910646107 20139300 PMC2840509

[B43] Schenk L. K. Bolger S. J. Luginbuhl K. Gonzales P. A. Rinschen M. M. Yu M. J. (2012). Quantitative proteomics identifies vasopressin-responsive nuclear proteins in collecting duct cells. *J. Am. Soc. Nephrol.* 23 1008–1018. 10.1681/ASN.2011070738 22440904 PMC3358758

[B44] Simon H. Gao Y. Franki N. Hays R. M. (1993). Vasopressin depolymerizes apical F-actin in rat inner medullary collecting duct. *Am. J. Physiol.* 265 C757–C762. 10.1152/ajpcell.1993.265.3.C757 8214031

[B45] Smythe E. Ayscough K. R. (2006). Actin regulation in endocytosis. *J. Cell Sci.* 119 4589–4598. 10.1242/jcs.03247 17093263

[B46] Star R. A. Nonoguchi H. Balaban R. Knepper M. A. (1988). Calcium and cyclic adenosine monophosphate as second messengers for vasopressin in the rat inner medullary collecting duct. *J. Clin. Invest.* 81 1879–1888. 10.1172/JCI113534 2838523 PMC442639

[B47] Sun T. X. Van Hoek A. Huang Y. Bouley R. McLaughlin M. Brown D. (2002). Aquaporin-2 localization in clathrin-coated pits: inhibition of endocytosis by dominant-negative dynamin. *Am. J. Physiol. Renal Physiol.* 282 F998–F1011. 10.1152/ajprenal.00257.2001 11997316

[B48] Tajika Y. Matsuzaki T. Suzuki T. Ablimit A. Aoki T. Hagiwara H. (2005). Differential regulation of AQP2 trafficking in endosomes by microtubules and actin filaments. *Histochem. Cell Biol.* 124 1–12. 10.1007/s00418-005-0010-3 16049696

[B49] Taylor A. Mamelak M. Reaven E. Maffly R. (1973). Vasopressin: possible role of microtubules and microfilaments in its action. *Science* 181 347–350. 10.1126/science.181.4097.347 4352609

[B50] Uawithya P. Pisitkun T. Ruttenberg B. E. Knepper M. A. (2008). Transcriptional profiling of native inner medullary collecting duct cells from rat kidney. *Physiol. Genomics* 32 229–253. 10.1152/physiolgenomics.00201.2007 17956998 PMC2276652

[B51] Uchida S. Matsumura Y. Rai T. Sasaki S. Marumo F. (1997). Regulation of aquaporin-2 gene transcription by GATA-3. *Biochem. Biophys. Res. Commun.* 232 65–68. 10.1006/bbrc.1997.6236 9125153

[B52] Wade J. B. Stetson D. L. Lewis S. A. (1981). ADH action: evidence for a membrane shuttle mechanism. *Ann. N. Y. Acad. Sci.* 372 106–117. 10.1111/j.1749-6632.1981.tb15464.x 6951416

[B53] Wang P. J. Lin S. T. Liu S. H. Kuo K. T. Hsu C. H. Knepper M. A. (2017). Vasopressin-induced serine 269 phosphorylation reduces Sipa1l1 (signal-induced proliferation-associated 1 like 1)-mediated aquaporin-2 endocytosis. *J. Biol. Chem.* 292 7984–7993. 10.1074/jbc.M117.779611 28336531 PMC5427275

[B54] Wang W. L. Su S. H. Wong K. Y. Yang C. W. Liu C. F. Yu M. J. (2020). Rab7 involves Vps35 to mediate AQP2 sorting and apical trafficking in collecting duct cells. *Am. J. Physiol. Renal Physiol.* 318 F956–F970. 10.1152/ajprenal.00297.2019 32088968

[B55] Wong K. Y. Wang W. L. Su S. H. Liu C. F. Yu M. J. (2020). Intracellular location of aquaporin-2 serine 269 phosphorylation and dephosphorylation in kidney collecting duct cells. *Am. J. Physiol. Renal Physiol.* 319 F592–F602. 10.1152/ajprenal.00205.2020 32799672

[B56] Xie L. Hoffert J. D. Chou C. L. Yu M. J. Pisitkun T. Knepper M. A. (2010). Quantitative analysis of aquaporin-2 phosphorylation. *Am. J. Physiol. Renal Physiol.* 298 F1018–F1023. 20089674 10.1152/ajprenal.00580.2009 PMC2853310

[B57] Yamamoto T. Sasaki S. Fushimi K. Ishibashi K. Yaoita E. Kawasaki K. (1995). Vasopressin increases AQP-CD water channel in apical membrane of collecting duct cells in Brattleboro rats. *Am. J. Physiol.* 268 C1546–C1551. 7541941 10.1152/ajpcell.1995.268.6.C1546

[B58] Yang C. R. Tongyoo P. Emamian M. Sandoval P. C. Raghuram V. Knepper M. A. (2015b). Deep proteomic profiling of vasopressin-sensitive collecting duct cells. I. Virtual Western blots and molecular weight distributions. *Am. J. Physiol. Cell Physiol.* 309 C785–C798. 10.1152/ajpcell.00213.2015 26310816 PMC4683217

[B59] Yang C. R. Raghuram V. Emamian M. Sandoval P. C. Knepper M. A. (2015a). Deep proteomic profiling of vasopressin-sensitive collecting duct cells. II. Bioinformatic analysis of vasopressin signaling. *Am. J. Physiol. Cell Physiol.* 309 C799–C812. 10.1152/ajpcell.00214.2015 26310817 PMC4683213

[B60] Yarar D. Waterman-Storer C. M. Schmid S. L. (2005). A dynamic actin cytoskeleton functions at multiple stages of clathrin-mediated endocytosis. *Mol. Biol. Cell* 16 964–975. 10.1091/mbc.e04-09-0774 15601897 PMC545926

[B61] Yasui M. Zelenin S. M. Celsi G. Aperia A. (1997). Adenylate cyclase-coupled vasopressin receptor activates AQP2 promoter via a dual effect on CRE and AP1 elements. *Am. J. Physiol.* 272 F443–F450. 10.1152/ajprenal.1997.272.4.F443 9140044

[B62] Yu M. J. Miller R. L. Uawithya P. Rinschen M. M. Khositseth S. Braucht D. W. (2009). Systems-level analysis of cell-specific AQP2 gene expression in renal collecting duct. *Proc. Natl. Acad. Sci. U.S.A.* 106 2441–2446. 10.1073/pnas.0813002106 19190182 PMC2650175

[B63] Yu M. J. Pisitkun T. Wang G. Aranda J. F. Gonzales P. A. Tchapyjnikov D. (2008). Large-scale quantitative LC-MS/MS analysis of detergent-resistant membrane proteins from rat renal collecting duct. *Am. J. Physiol. Cell Physiol.* 295 C661–C678. 10.1152/ajpcell.90650.2007 18596208 PMC2544440

[B64] Yui N. Lu H. J. Bouley R. Brown D. (2012). AQP2 is necessary for vasopressin- and forskolin-mediated filamentous actin depolymerization in renal epithelial cells. *Biol. Open* 1 101–108. 10.1242/bio.2011042 23213402 PMC3507199

[B65] Zhao X. Khurana S. Charkraborty S. Tian Y. Sedor J. R. Bruggman L. A. (2017). alpha Actinin 4 (ACTN4) regulates glucocorticoid receptor-mediated transactivation and transrepression in podocytes. *J. Biol. Chem.* 292 1637–1647. 10.1074/jbc.M116.755546 27998979 PMC5290941

